# Comment #2 on “First-Line *Helicobacter pylori* Eradication with Vonoprazan, Clarithromycin, and Metronidazole in Patients Allergic to Penicillin”

**DOI:** 10.1155/2018/5260358

**Published:** 2018-12-16

**Authors:** Davide Giuseppe Ribaldone, Marco Astegiano

**Affiliations:** ^1^Department of Medical Sciences, Division of Gastroenterology, University of Turin, C.so Bramante 88, 10126 Turin, Italy; ^2^Department of General and Specialist Medicine, Gastroenterology-U, Città della Salute e della Scienza di Torino, Italy

Recently, Sue et al. published an open-label study, aiming to assess the efficacy of a 7-day first-line *Helicobacter pylori* (*H. pylori*) eradication regimen with vonoprazan (VPZ), clarithromycin (CAM), and metronidazole (MNZ), in patients with penicillin allergy [1]. In a letter to the editor, Kashani and Abadi raised several criticisms of this article [2]. The authors responded [3], but we want to focus on two not redundant points.

Considering appropriate some considerations (already reported by Sue et al.), especially those of the lack of controls and of the small sample size, we do not agree with two points. First, in the letter, it is reported that the factors affecting the success rate of *H. pylori* therapy were not checked and, among those, smoking habits and alcohol-drinking habits were reported. About these factors, there is no universal agreement in the literature [4, 5] and guidelines [6] on their ability to predict a poor response.

Second, Kashani and Abadi reported the need to evaluate CAM and MNZ resistance before deciding the appropriate treatment [2]. This is correct [7]; however, it should be highlighted that it is possible to obtain *H. pylori* eradication with VPZ-based therapy in 70.2% of patients in whom rabeprazole-based therapy (with the same antibiotics) has failed [8]. Thus, VPZ-based treatment shows a relatively high eradication rate against clarithromycin-resistant *H. pylori.* A plausible explanation is that, since VPZ and CAM are metabolized by CYP3A4, a combined treatment with these three drugs can delay their clearance permitting a prolonged and more potent effect. In addition, the strong and fast-acting acid inhibitory effect of VPZ allowed the antibiotics to eradicate *H. pylori* [9]. Nevertheless, the efficacy of VPZ has not been reported for the combination of VPZ, CAM, and MNZ. Regarding the comment of Kashani and Abadi on the need to evaluate MNZ resistance by susceptibility tests, we would highlight that this resistance, although highly prevalent, can be partly overcome and is of secondary importance. Hence, the need to evaluate MNZ resistance patient-by-patient could not be useful. It would be better to know in a specific population MNZ and CAM resistance rates and apply the recommendation of the more updated guidelines [6].
